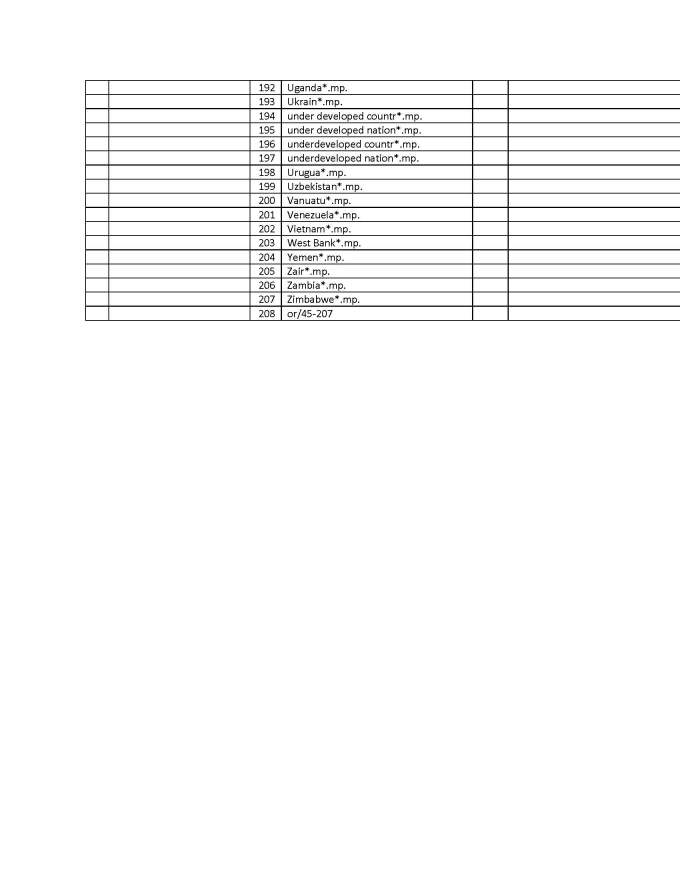# Earthquakes to Floods: A Scoping Review of Health-related Disaster Research in Low- and Middle-income Countries

**DOI:** 10.1371/currents.dis.57d98a902a326361d88d54521e68b016

**Published:** 2018-08-30

**Authors:** Catherine M. Tansey, John Pringle, Anushree Davé, Renaud Boulanger, Matthew Hunt

**Affiliations:** Humanitarian Health Ethics Research Group, McGill University, Montréal, Québec, Canada and McMaster University, Montréal, Québec, Canada; Assistant Professor at the Ingram School of Nursing, McGill University, Montréal, Québec, Canada; Humanitarian Health Ethics Research Group, McGill University, Montréal, Québec, Canada and McMaster University, Hamilton, Canada; McGill University Health Centre; Centre for Interdisciplinary Research in Rehabilitation, School of Physical and Occupational Therapy, McGill University, Montréal, Québec, Canada

## Abstract

Introduction: Health-related disaster research is a relatively small; but growing field of inquiry.  A better understanding of the scope and scale of health-related disaster research that has occurred in low- and middle-income countries (LMICs) would be useful to funders, researchers, humanitarian aid organizations, and governments as they strive to identify gaps, disparities, trends, and needs of populations affected by disasters.

Methodology: We performed a scoping review using the process outlined by Arksey & O’Malley to assess the characteristics of peer-reviewed publications of empirical health-related disaster research conducted in LMICs and published in the years 2003-2012.

Results: Five hundred and eighty-two relevant publications were identified.  Earthquakes were by far the most commonly researched events (62% of articles) in the review’s timeframe.  More articles were published about disasters in China & South Asia/South East Asia than all other regions.  Just over half of the articles (51%) were published by research teams in which all the authors’ primary listed affiliations were with an institution located in the same country where the research was conducted.  Most of the articles were classified as either mental health, neurology and stress physiology (35%) or as traumatology, wounds and surgery (19%).  In just over half of the articles (54%), data collection was initiated within 3 months of the disaster, and in 13% research was initiated between 3 and 6 months following the disaster.  The articles in our review were published in 282 different journals.

Discussion: The high number of publications studying consequences of an earthquake may not be surprising, given that earthquakes are devastating sudden onset events in LMICs.  Researchers study topics that require immediate attention following a disaster, such as trauma surgery, as well as health problems that manifest later, such as post-traumatic stress disorder.  One neglected area of study during the review’s timeframe was the impact of disasters on non-communicable and chronic diseases (excluding mental health), and the management of these conditions in the aftermath of disasters. Strengthening disaster research capacity is critical for fostering robust research in the aftermath of disasters, a particular need in LMICs.

## Introduction:

Disaster research endeavours to increase our understanding of disasters and their consequences for human health and societies, as well as to develop evidence that will lead to improved emergency responses. Health-related disaster research is a relatively small field of inquiry, but it is growing.^1^ By health-related disaster research, we are referring to “studies conducted with human subjects during or following an event such as an earthquake, flood, hurricane, tsunami or typhoon”^2^. However, there is currently limited knowledge about the nature and scope of health-related human subject research implemented in disasters, particularly of those studies conducted in low- and middle-income countries (LMICs). A better understanding of the scope and the scale of health-related disaster research that has occurred in LMICs would be useful to responders, funders, researchers, research institutions, and governments as they seek to identify gaps, disparities, trends and needs of populations affected by disasters. Disasters are increasing worldwide^3^ and they occur disproportionately in LMICs^4^ where there are fewer resources for disaster preparedness and response and often less capacity to undertake research.

As interest in disaster research grows, so too does the number of journals dedicated to this area of inquiry. Many disaster-related articles are published in non-disaster specific journals in fields such as medicine, public health, geography, development studies, anthropology, and history. However, as shown by several syntheses of this literature^5^^, ^6^, ^7^, ^8^, ^9 , many of these articles are not original empirical research articles. We therefore conducted a scoping review of articles reporting health-related disaster research conducted in LMICs and published between 2003 and 2012. We chose to make LMICs our area of focus since “85% of disasters and 95% of disaster-related deaths occur in the developing world”^5^. To our knowledge, no review to date has focused exclusively on original research publications of studies conducted during or in the aftermath of disasters in LMICs.

## Methodology

The scoping review followed the five-step process described by Arksey & O’Malley^10^ and incorporated several refinements proposed by Levac et al.^11^. These included holding team meetings at the inception, midpoint and end of the article review process to discuss challenges and uncertainties and the selection of variables to extract from selected articles.

The primary research question was the following: What are the characteristics of empirical health-related disaster research in low-and-middle-income countries (LMICs)? A second line of inquiry examined how ethics procedures and ethical issues are reported in the disaster literature, and these findings are reported elsewhere.^12^

We focused on climate-related and geophysical events that occurred in LMICs and included situations of natural hazards (excluding situations such as war, epidemics and technological disasters) that are listed in the National Library of Medicine’s Medical Subject Headings (MESH). To define an LMIC we used the World Bank classification as it stood in 2012 i.e., at the inception of this project.^13^

A key-word search was conducted in 2013 (see appendix 3 for strategy used in Ovid Medline) with the help of a librarian to generate a comprehensive list of potentially relevant articles. Using Ovid Medline and Embase databases to identify health-related disaster research, a list of 10,154 citations was generated

Inclusion criteria to be retained for review were as follows:

1. The study was initiated during or within 2 years following a natural disaster;
2. The study was conducted in a LMIC;
3. The study involved living human participants (studies of human remains were excluded);
4. The study involved the systematic collection of data;
5. The article was published between 2003-2012 inclusively;
6. The article was in English, French, or Spanish;
7. The full-text of the publication was retrievable through the McGill University Library Services or via its inter-library loan system.

We excluded condensed publications such as abstracts or conference proceedings, as well as single case studies or case reports.

A total of six reviewers were involved in reading the abstracts in the initial phase, with every citation assessed independently by two people. When there was no abstract or when there was insufficient information in the abstract to determine if the study met the inclusion criteria, the full article was reviewed. Disagreements between reviewers were resolved through discussion with at least one other reviewer. The authors of 65 articles did not specify when data collection began. While these articles may not explicitly have met our inclusion criteria, the reviewers judged that the start time was most likely within the time frame that we were looking at (i.e., within two years of the disaster event). Screening for duplicates was carried out several times. From the 10,154 citations, 582 articles were deemed eligible and were retained for analysis and the list of these articles is available at: https://figshare.com/s/f3d907a3515dcdcaa4c8.

**Figure:**
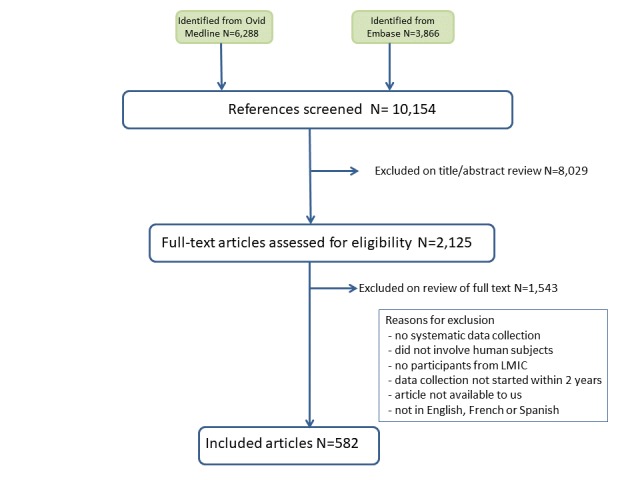
Figure 1 - PRISMA Flow Chart

The research team collaborated to develop the data extraction scheme. The choice of variables and the format in which they were collected was refined as the review progressed. Twenty-six variables, ranging from the type of disaster and country where it occurred, to the topic of the research, were entered into a Microsoft Access 2016 database. Of these, we present findings for eight key variables: type of disaster, location of disasters, publication by year, authorship, research topics, initiation of data collection, research design, and journals in which the study was published. We used univariate analysis (primarily frequencies) of key descriptor variables and multivariate analysis to examine possible relationships between variables. No significance testing was done, as none was planned a priori. Microsoft Excel 2016 was used for the analysis.

## Results

Type of Disaster

The articles identified in this review pertain to 12 disaster types and report on research that was conducted in 43 LMICs from 2003 to 2012. Earthquakes were by far the most commonly researched events in the review’s timeframe; 359 of 582 articles (62%) reported on research conducted following this type of disaster. When tsunamis, which are usually caused by undersea earthquakes, are included with this figure the number rises to 464 articles (80% of the publications). Two events were largely responsible for the prominence of earthquake and tsunami research in the review: the 2008 Sichuan earthquake (n=153, 26% of all articles), and the 2004 Indian Ocean tsunami (n=104, 18%). The 2010 Haiti earthquake (n=40, 7%), the 2003 Bam earthquake (n=37, 6%), the Pakistan/Kashmir earthquake in 2005 (n=33, 6%) and the 1999 earthquakes in the Marmara region of Turkey (n=33, 6%) also figured prominently. Table 1 shows the distribution of research articles by disaster type.

**Figure:**
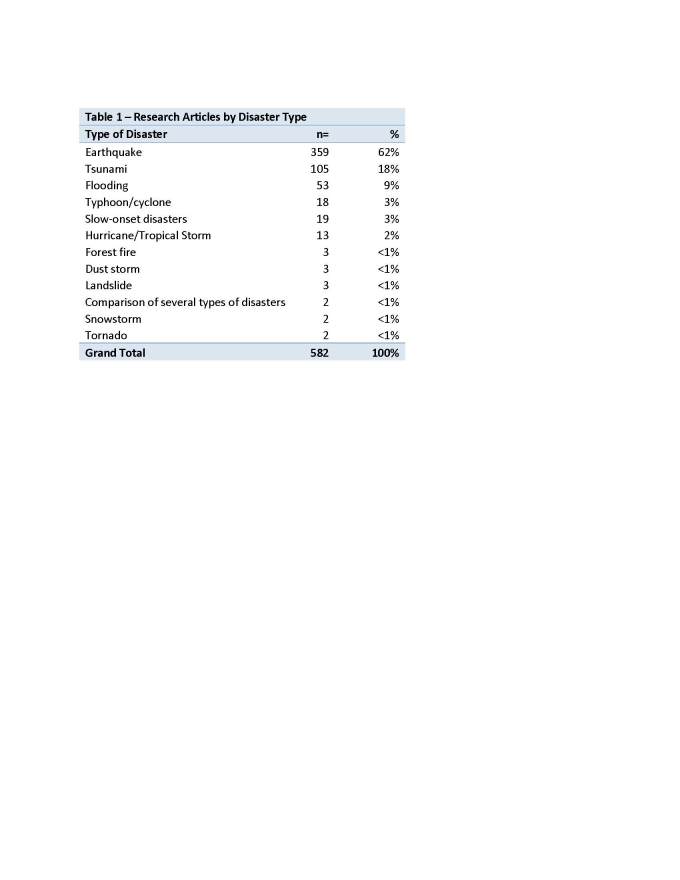

Geographic Distribution of Disasters

**Figure:**
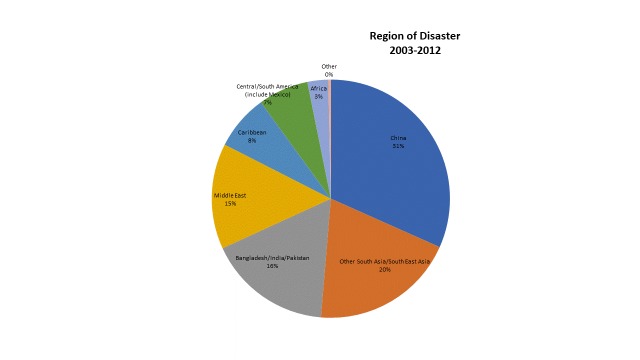
Figure 2 - Regions of Disaster Research

Over 50% of the publications reported on research conducted in China & South Asia/South East Asia (Figure 2). This finding was driven by the many publications about the 2008 Sichuan earthquake and the Indian Ocean tsunami.

The geographical pattern changes markedly over the course of the review’s timeframe, depending on which large disaster occurred in the previous years. For example, in 2003, 54% of the publications (15/28) pertained to the 1999 earthquakes in Turkey. In 2008, 71% of the articles (25/35) pertained to countries in South Asia/South East Asia including Bangladesh/India/Pakistan and primarily discussed aspects of the 2004 tsunami (49%), and the 2003 earthquake in Iran (17%).

Publication by Year

Research publications show an increasing trend (line of best fit) over time as illustrated by the graph (figure 3) of the number of publications per year.

**Figure:**
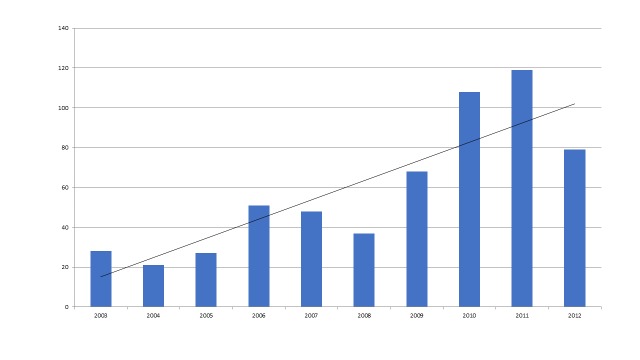
Figure 3 - Disaster Research Publications by Year

The trend is driven by the two most devastating (i.e., large number of deaths) disasters experienced over the period of our review: the Sichuan earthquake and the Indian Ocean tsunami. However, even when we remove publications about the Sichuan earthquake and the 2004 tsunami, the trend remains, albeit less obvious. (graph not shown)

Depicting the data in a different way (figure 4), we can see trends in publication frequency by disaster type. Some disaster types (e.g., hurricane/tropical storms and floods) show relatively stable rates of publication over the ten-year period. Others spike when major disaster events with large mortality rates occur, such as the 2004 tsunami, the 2008 Sichuan earthquake and the 2010 Haiti earthquake. Due to the lag between an event and when research on that event is published, spikes in publication occur in the years following the corresponding disasters.

**Figure:**
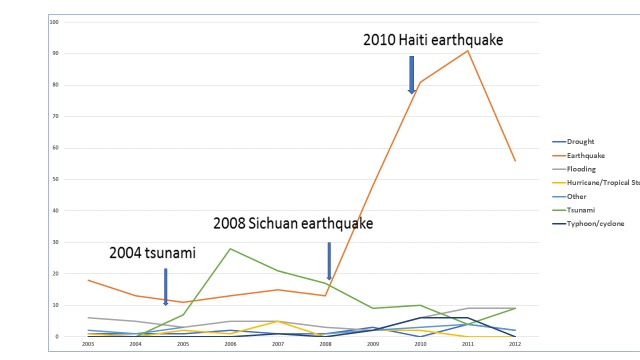
Figure 4 - Type of Disaster/Year Published

While the Haiti earthquake (n=40) was in the timeframe that we investigated, many publications concerning this event may have appeared after our end date of 2012. As depicted in the graph below (figure 5) which shows publications from the Sichuan earthquake which occurred in May 2008, there is a significant lag between the occurrence of the disaster and when research is published. Of note, there are no publications about this event in 2008 i.e., in the first six months after the event.

**Figure:**
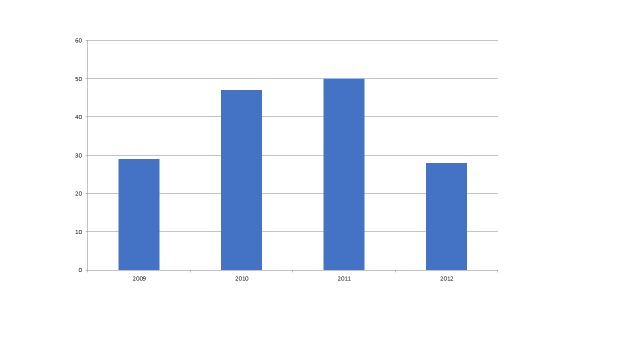
Figure 5 - Publications about 2008 Earthquake

Similarly, publications about the 2004 Indian Ocean tsunami are spread over at least 7 years and show a time lag to first publication, with just 7 articles appearing in 2005. (Figure 6)

**Figure:**
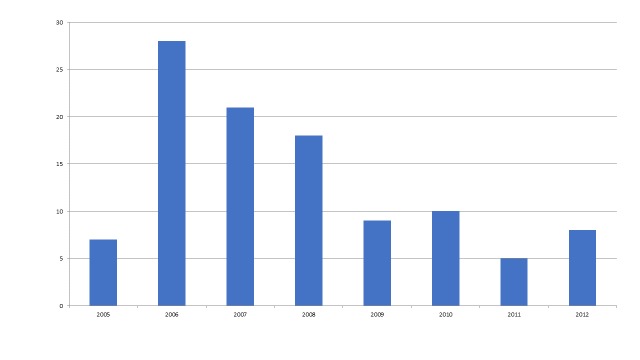
Figure 6 - Publications/Year about the 2004 Indian Ocean Tsunami

Authorship

A very high proportion of author teams identified their primary affiliations as being with academic institutions or hospitals (492/582=85%). Seventy-one articles (12%) were authored by teams that included academic and/or hospital-based researchers collaborating with researchers affiliated with governments, militaries or non-governmental organizations (NGOs). Relatively few teams of authors were composed of researchers affiliated solely with governments (n=9), militaries (n=5), or NGOs (n=3). In 2 articles, author affiliations were not disclosed.

Just over half of the articles (296/582=51%) were published by research teams in which all the authors’ primary listed affiliations were with an institution located in the country where the research was conducted. A major driver of this high rate of research produced by national research teams was publications pertaining to the 2008 Sichuan earthquake. Almost 78% (120/153) of articles related to this event were published by teams consisting exclusively of researchers based in China. This high level of research conducted by national researchers contrasts with research pertaining to the 2010 Haiti earthquake where none of the 40 publications was written by a research team in which all members had their primary affiliation at a Haitian institution. In fact, 30 of the 40 publications identified were authored without any Haiti-based collaborators. Articles pertaining to the 2004 Indian Ocean tsunami fell between these two with 36/104=35% of the research teams being based exclusively in the country where the research was conducted.

Twenty-two percent (128/582) of papers were authored by teams exclusively from a country other than where the disaster occurred. Most often, the first author of these papers was based at an institution in North America (71/128=55%) (6 Canada, 65 US).

Research Topics

Over half of the articles (320/582=55%) pertained to mental health, neurology and stress physiology (35%) or traumatology, wounds and surgery (19%). The number of articles published by research topic is listed below (table 2).

**Figure:**
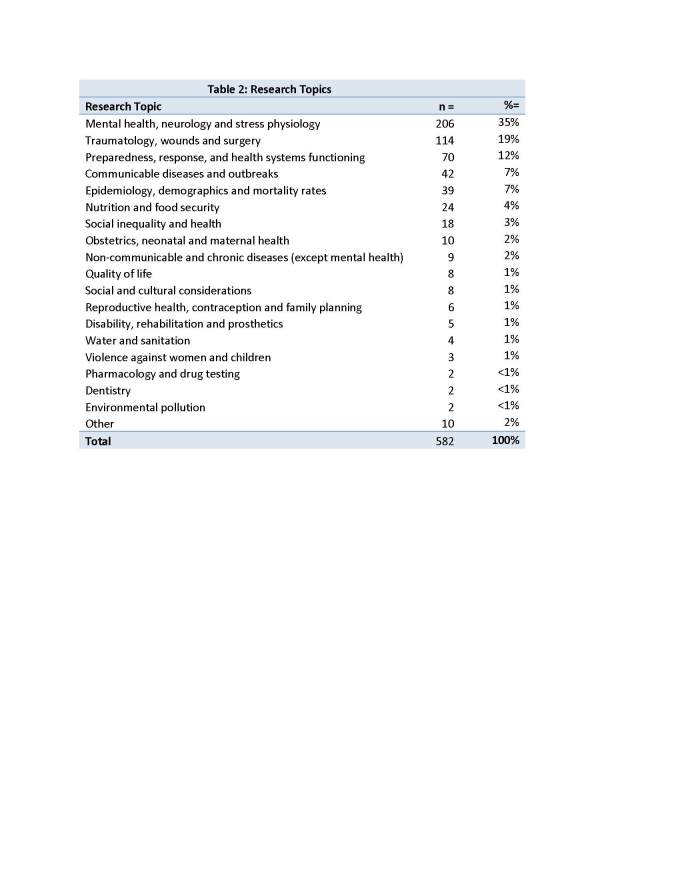

Table 3 and figure 7 compare the various disaster types with the major topics covered.

**Figure:**
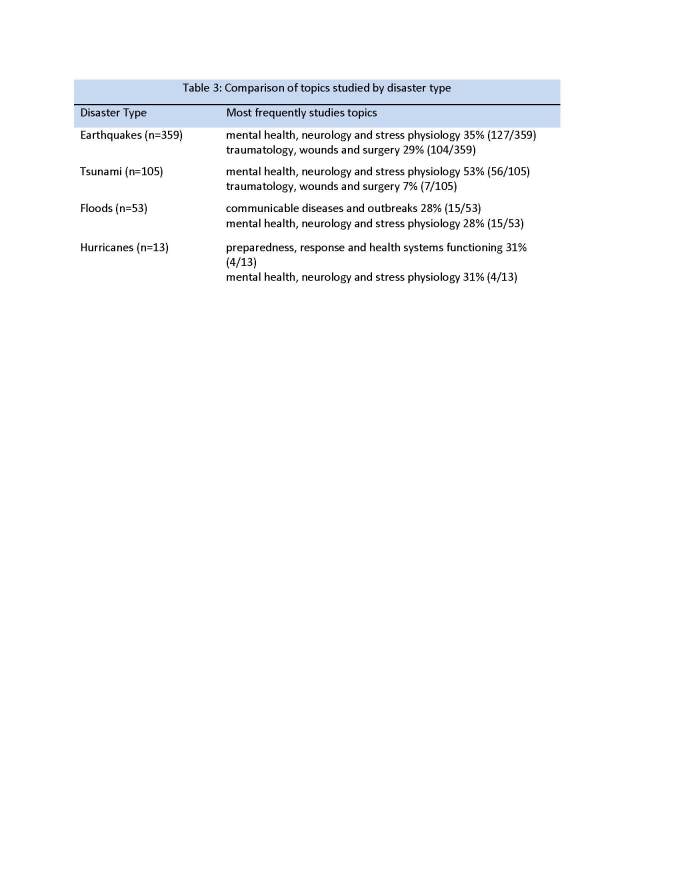

**Figure:**
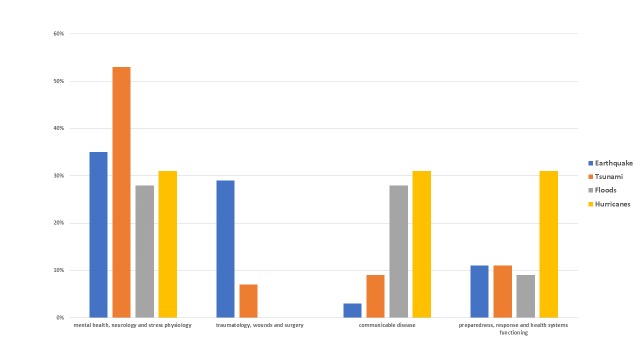
Figure 7 - Comparison of Topics Studied by Disaster Type

Initiation of Data Collection

In just over half of the articles (312/582=54%), data collection was started within 3 months of the disaster, and in 73/582=13% it was initiated between 3 and 6 months following the disaster. As depicted below (figure 8), relatively few studies were initiated later than 6 months after the event. Early initiation of research was most marked in traumatology, wound and surgery studies where 82/114=72% of studies initiated data collection within 3 months after the acute event. For mental health, neurology and stress physiology studies (n=206), there was a slightly greater percentage of studies started later (≤ 3 months=84/206=41% and 3-6 months after=41/206=20%) compared to the articles overall.

**Figure:**
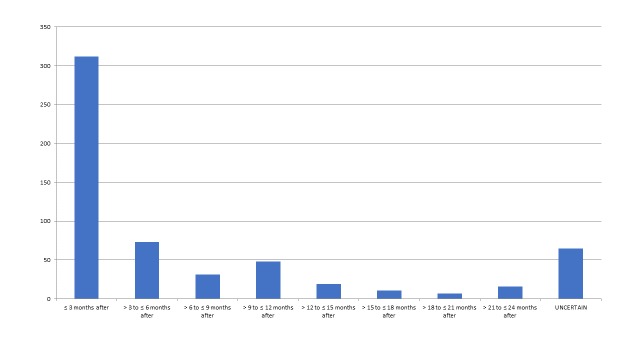
Figure 8 - Initiation of Data Collection

Research Design

Forty-eight percent (281/582) of the studies used a cross-sectional design and 17% (97/582) were retrospective analyses of data that was previously collected for non-research purposes, such as clinical care. Other study designs are listed below (table 4). In 12 of the studies (2%), the methods were not described clearly enough for us to classify the study design.

The high prevalence of cross-sectional methods is observed across all disaster types. However, in hurricane and tsunami related studies, qualitative and mixed methods were used more frequently, and chart reviews not as often, compared to the articles overall. Qualitative or mixed methods were used in 38% of hurricane and 26% of tsunami studies.

In table 5, we show the frequency with which different methods were used to investigate the three most commonly studied topics: mental health, neurology and stress physiology; traumatology, wounds and surgery; preparedness, response, and health systems functioning. We note that cross-sectional studies figure prominently in all topics, but that chart reviews were not used in mental health, neurology and stress physiology studies.

**Figure:**
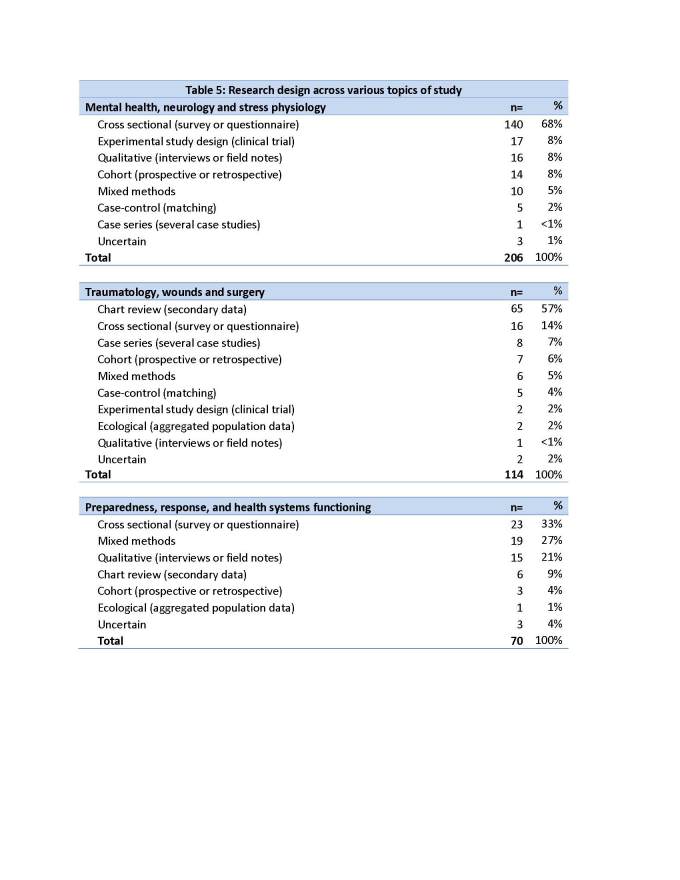

Of the 582 articles, 22 report a clinical trial. Eight of the trials began during the first three months after the disaster and another 7 trials were initiated 3 to 6 months after the event, with the remaining 8 studies beginning data collection later than 6 months after the disaster. Fourteen of these studies were conducted after earthquakes and 6 after the Indian Ocean tsunami. Seventeen of the 22 articles about clinical trials investigated interventions in the areas of mental health, neurology and stress physiology. Fifteen of the 22 articles (68%) were conducted by teams exclusively made up of researchers from the country of the disaster.

Journals

The articles in our review were published in 282 different journals. One hundred and nine (19%) articles were published in the 5 most frequently chosen journals: Prehospital & Disaster Medicine, Disasters, Journal of Traumatic Stress, PLoS One, and Injury. These five journals are international in scope, are based in the US or Europe and their 2012 impact factors ranged from 0.9 (Disasters) to 3.2 (PLoS One).

## Discussion

Type of disaster

Our scoping review found that health-related disaster research in LMICs published from 2003-2012 most frequently pertained to earthquakes (62% of articles), a finding we did not expect. Because the Sichuan earthquake was so devastating, and studied so frequently by Chinese researchers, our review contained many studies written by Chinese authors, another unexpected finding. A review of Iranian disaster publications by Babaie and colleagues^14^ similarly found that the greatest proportion (29%) of their disaster studies were conducted after an earthquake. The difference in proportion of earthquake-related research between our review and Babaie and colleagues’ is likely due to the inclusion of events that are not geophysical or meteorological in the latter review. In retrospect, the high prevalence of publications studying consequences of an earthquake in the two reviews may not be surprising, given that earthquakes are especially deathly events in LMICs. The EM-DAT database of international disasters shows that major earthquakes are amongst the events with the highest mortality rates and the greatest human impact (table 6). While droughts also affect millions of people, attributed mortality rates are lower in comparison. This difference may explain why we found few articles about droughts (n=16), and none about the large drought in South Africa in 2004.

**Figure:**
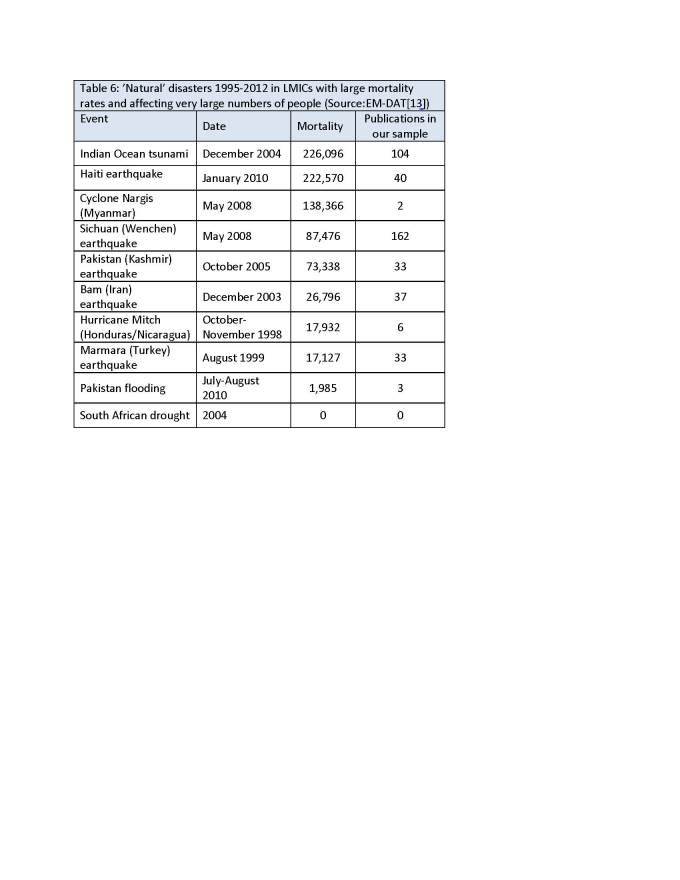

We have plotted the number of publications in our study against the number of reported deaths in the disaster events (Figure 9). Visually, we see that two events are far from the line of best fit: the Sichuan earthquake which is over represented in our sample and Cyclone Nargis which has many fewer publications than we would have expected. Similarly, when we plot number of publications against total damage (data not shown), Cyclone Nargis again has fewer publications than expected, but the Indian Ocean tsunami has more publications that would be predicted. It must be noted that these are imperfect measures given how long after the event publications continue to occur.

**Figure:**
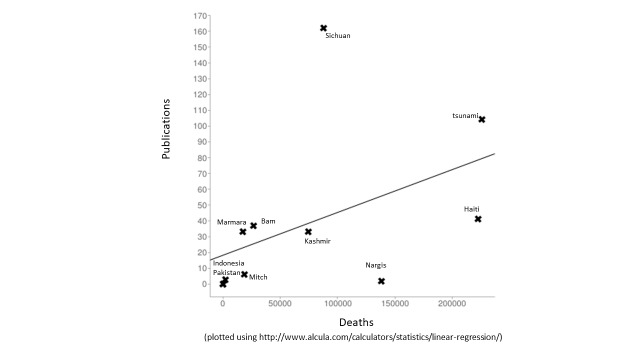
Figure 9 - Number of Publications versus Number of Deaths due to a Disaster Event

Geographic Distribution of Disasters

During the time period of this review, several disasters also occurred in South America (e.g., earthquake in Peru in 2007). However, because they occurred in locales that were not as heavily populated as the disaster areas in Asia, they resulted in lower morbidity and mortality rates and perhaps this is why few research articles were identified in our study pertaining to these events. Studies about South American events may have been published in Spanish journals that are not included in Ovid Medline or Embase. In their review of disaster-specific literature from 1977–2009, Smith et al.^9^ also found that there were more research publications following high impact events that received extensive media attention (e.g., 11 September 2001, Indian Ocean earthquake/ tsunami, and Hurricane Katrina) compared to events that were less devastating and/or that received less media coverage. We found few publications from Africa which could be because the continent is less prone to earthquakes (http://gmo.gfz-potsdam.de/)^15^; no large earthquake occurred on the continent in the timeframe that we investigated. Other events in this timeframe which led to millions of displaced persons in Africa were the result of civil strife (e.g., in Sudan) and, as such, were excluded from our review.

Publication by Year

We have shown increases in publication rates of health-related disaster research from 2003-2012 and have linked this trend to a set of particularly devastating geophysical events, including the 2004 Indian Ocean tsunami, the 2008 Sichuan earthquake, and the 2010 Haiti earthquake. However, other factors should be taken into consideration when extrapolating the growth of health-related disaster research publications, such as the increase in scientific publications overall^16^.

On the one hand, one humanitarian organization Médecin Sans Frontières reports that research is a growing focus of the organization and that this trend is likely to continue at least within their organization^17^. We can expect to see an increase in disaster research pertaining to climate-change and ecological collapse^18^. Urbanization and increased habitation of flood plains and coastal areas will leave more individuals at risk. This may translate into more publications about the health-related effects of disasters.

On the other hand, we show fewer publications in 2012 as compared to the previous 2 years. It is unclear if this is a spurious result with some publications not yet indexed at the time of the literature search. It is possible that there is a surge in research with each large disaster and a subsequent reversion to baseline. Thus, we are unable to speculate whether the trend of increasing number of health-related disaster publications is real and whether it will hold into future years.

Lag in publication from the date of the event

We have shown a time lag from the date of the disaster event until the first research publications appear. We postulate that this lag time represents the time it takes researchers to develop their protocol, obtain research ethics approval, conduct the study, analyse the data, write and submit a manuscript, and have it published. The journal PLoS Currents Disasters has been developed to help increase capacity for disaster-related publications and to lessen the time from completion of a study to its publication date. Pre-planned ‘generic’ protocols may also help to reduce this lag^2^.

Authorship

Investigators from some LMICs were much more likely to lead research studies related to disasters occurring in their own country. In particular, we found that Chinese and Iranian researchers published frequently about the disasters that occurred in their countries. This pattern may reflect the fact that they are upper-middle income countries with strong central governments that have more capacity for research than many low-income-countries such as Haiti. In contrast to the Chinese and Iranian publication rates noted in our study, Roy et al.^5^ reported that only 20% of authors in their sample of publications about disasters which occurred in the developing world were written by authors with affiliations in the developing world. This may be explained by the methodological differences between the two studies (they included commentaries and reviews).

One area that deserves further study is how the location of a disaster within a country influences the extent to which national or international researchers undertake research. We speculate that in remote settings, national researchers may be more likely than international researchers to gain access for research. In contrast, in situations where the disaster is more widespread or directly affecting the location where researchers are situated, national researchers may be less able to mobilize. For instance, the 2010 Haiti earthquake was centered in the capital city of Port-aux-Prince, where most universities and researchers are situated. In a context where research capacity was already limited, the location and scale of the disaster would have made it more difficult for local researchers to undertake research when many university buildings were badly damaged and/or destroyed (http://www.nytimes.com/2010/02/14/world/americas/14schools.html), and the death toll was extremely high.

Research Topics

By some definitions, mental health falls under the category of non-communicable chronic diseases (NCD)^19^ and it is the one NCD that was often the subject of research in our review. It is also an area of research that is culturally sensitive, and interventions must be tailored to the locale, which may generate context-specific manuscripts. This high prevalence of mental health research that we found is in contrast to that reported by Roy et al.^5^. They found that just 4.2% of the articles in their sample related to mental health. Instead, their sample found that 30% of articles were focused on mission and policy. Here too, the difference may be explained by the fact that they included commentaries and reviews in their study that were excluded from our sample.

Articles about other NCDs, however were few in our scoping review (9 out of 582). Other authors have noted this phenomenon and have called for greater attention to the needs of those with chronic conditions in the aftermath of a disaster. In fact, Demaio et al. “call on all sectors to recognise and address the specific health challenges posed by non-communicable diseases in emergencies and disaster situations”^20^. Particularly important are the needs of those requiring daily medication which must be refrigerated (e.g., insulin) and those requiring technological support (e.g., dialysis). One study conducted in China after the Sichuan earthquake found that 77% of patients presenting to a clinic in the acute phase of the recovery required urgent medical attention for a chronic disease.^21^ Diabetes is a good example of a chronic illness that can be affected by a disaster. Much was written about the management of diabetes after Hurricane Katrina, and lessons can be learned from that experience.^22^^, ^23 In LMICs, the extent of this problem can be expected to increase as populations age. For instance, Baruah et al.^24^ comment that diabetes is of particular concern in India where it is estimated that by 2030 the country will have the largest number of diabetics in the world. In their study of the 2001 Gujarat earthquake, the authors noted that “lack of health information cards, stress induced hyperglycemia, mismatch between the medical supplies and expired medications” were problematic for diabetics.^24^

Research Design

Because our review focused on primary research, the proportions that we report for various study designs are not comparable to those found in other reviews^6^^, ^25. Despite this, we see that cross-sectional designs figure prominently in several reviews with both Abramson et al.^6^ and Savoria et al.^25^ reporting it as the most common research design after review/commentary.

Clinical trials (22 in our sample) deserve special mention since they can be the riskiest and the most resource-intensive. Despite this, 17 (77%) of the clinical trials were initiated during the first six months after the disaster. Further establishing the primacy of mental health research after a disaster, we found that 18 (82%) of the clinical trials were in this area. Recently, a Lancet Series on health in humanitarian crises^26^ has highlighted the need for “a stronger evidence base to improve the effectiveness and efficiency of humanitarian actions”^26^. An article by Blanchet et al.^27^ that discusses public health research during humanitarian crises notes a dearth of experimental designs able to infer causality.

Limitations

Our review has several limitations. First, we searched only Ovid Medline and Embase databases. This choice reflects our focus on health-related disaster research. By adding additional databases from the social sciences and humanities, we may have identified additional articles.

While the number of journals listed in Ovid Medline and Embase is very large, they tend to be journals with an international focus. Articles tend to be in English or have English abstracts. Smith et al.^9^ compared the number of publications retrieved about the Bhopal disaster using Medline compared to several other databases (EMBASE, CINAHL, AMED, CENTRAL, Psych Info, Maternity and Infant Care, EBM Reviews) and found that seventy-five percent of articles retrieved were also found in Medline. While this is reassuring for our search, the results might have been different for a disaster in a country whose primary language is not English and with databases that include articles without an English abstract. This was demonstrated by the authors of the summary of Iranian disaster studies^14^ who retrieved 389 articles while searching several databases that list Iranian journals. We found only 39 publications from Iran. Even though they searched a longer time frame (varying depending on the database), it is likely that we failed to capture some articles that were published during our period of inclusion. We limited publication languages to those understood and read by our team members (English, French and Spanish), so research publications in other languages are not represented in our findings.

## Conclusion

Our scoping review assessed the characteristics of peer-reviewed publications of empirical health-related disaster research conducted in LMICs and published in 2003-2012. To our knowledge, this is the first synthesis to focus exclusively on health-related empirical research conducted during disasters. We found that many health-related research publications focus on the most devastating events, namely those that affect the largest number of people and those with the highest mortality rates. The most common research topics were those requiring the most immediate attention, such as traumatology, wounds, and the resultant surgeries typically carried out within hours or days of a disaster. In the months and years following a disaster, research attention turned to the mental health sequelae such as post-traumatic stress and depression. Three main recommendations are apparent from this study: 1) the need to increase post-disaster health-related research capacity particularly in LMICs; 2) the need to research chronic diseases and their management after a disaster and 3) the need for methodologically rigorous research using a wide range of study designs, including ones that can produce strong evidence to guide practice and policy, and which go beyond cross-sectional descriptive studies.

## Corresponding Author

Matthew Hunt, PhD

## Competing Interests

The authors have read PLOS Currents policy and have no competing interests to declare.

## Data Availability Statement

A list of the 582 selected articles is found in https://figshare.com/s/f3d907a3515dcdcaa4c8

## Appendix 1 – list of 582 articles included

A list of the 582 selected articles is found in Appendix 1 at: https://figshare.com/s/f3d907a3515dcdcaa4c8

## Appendix 2 – list of disasters included

animal infestations

ash fall

avalanche

blizzard

cyclone

drought

dust storm

earthquake

erosion

extreme cold

extreme heat

fire

flood

hail storm

heatwave

hurricane

ice storm

insect infestation

landslide

lava flow

mudslide

plant disease

sandstorm

thunderstorm

tornado

tropical storm

tsunami

typhoon

volcano

wildfire

## Appendix 3 - Search Strategy in Ovid

**Figure:**
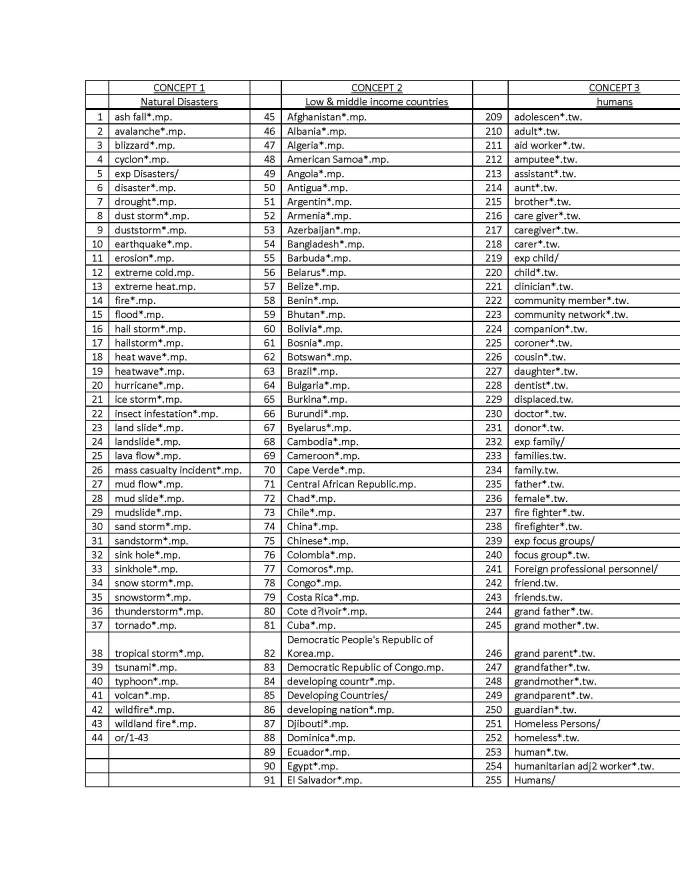

**Figure:**
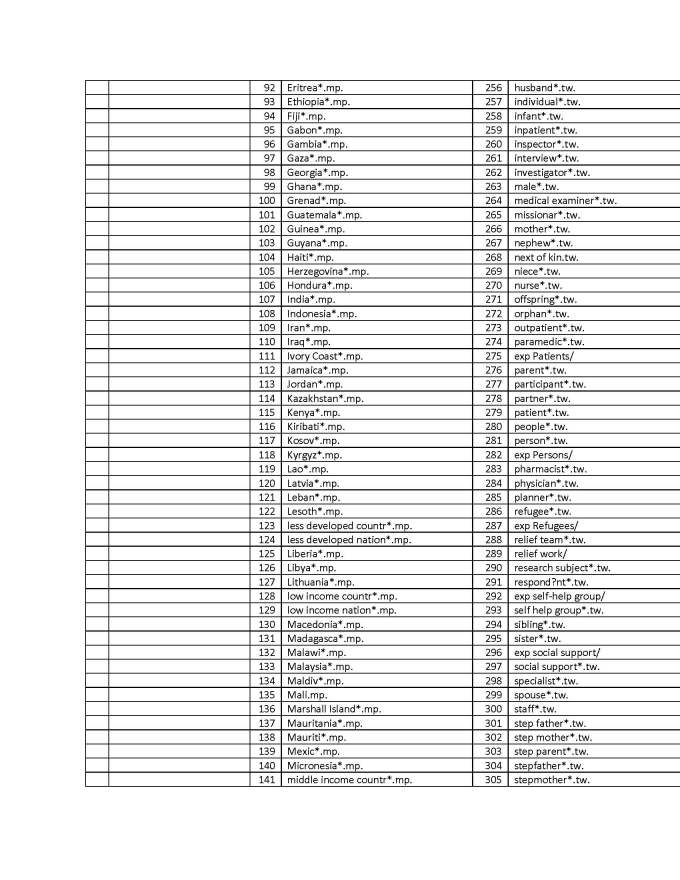

**Figure:**
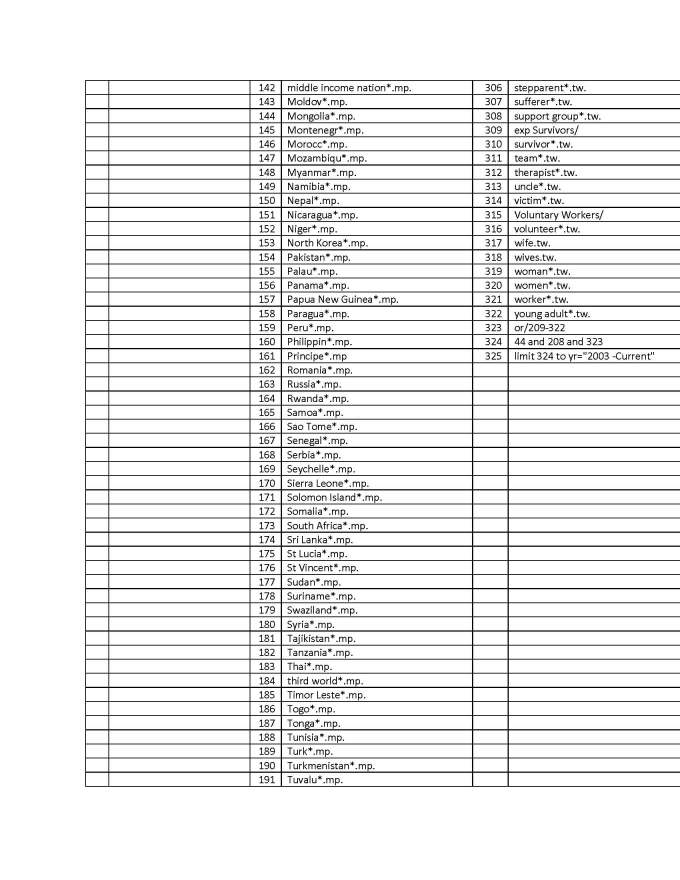

**Figure:**